# A Motivational Determinant of Facial Emotion Recognition: Regulatory Focus Affects Recognition of Emotions in Faces

**DOI:** 10.1371/journal.pone.0112383

**Published:** 2014-11-07

**Authors:** Claudia Sassenrath, Kai Sassenberg, Devin G. Ray, Katharina Scheiter, Halszka Jarodzka

**Affiliations:** 1 Knowledge Media Research Center, Social Processes Lab, Tübingen, Germany; 2 University of Ulm, Department of Social Psychology, Ulm, Germany; 3 University of Tübingen, Department of Psychology, Tübingen, Germany; 4 University of Aberdeen, School of Psychology, Aberdeen, United Kingdom; 5 Open Universiteit Nederland, Center for Learning Sciences and Technologies, Heerlen, The Netherlands; University of Gent, Belgium

## Abstract

Two studies examined an unexplored motivational determinant of facial emotion recognition: observer regulatory focus. It was predicted that a promotion focus would enhance facial emotion recognition relative to a prevention focus because the attentional strategies associated with promotion focus enhance performance on well-learned or innate tasks - such as facial emotion recognition. In Study 1, a promotion or a prevention focus was experimentally induced and better facial emotion recognition was observed in a promotion focus compared to a prevention focus. In Study 2, individual differences in chronic regulatory focus were assessed and attention allocation was measured using eye tracking during the facial emotion recognition task. Results indicated that the positive relation between a promotion focus and facial emotion recognition is mediated by shorter fixation duration on the face which reflects a pattern of attention allocation matched to the eager strategy in a promotion focus (i.e., striving to make hits). A prevention focus did not have an impact neither on perceptual processing nor on facial emotion recognition. Taken together, these findings demonstrate important mechanisms and consequences of observer motivational orientation for facial emotion recognition.

## Introduction

Faces are amongst the most relevant social stimuli as they communicate information essential for the course of social interaction and communication. Specifically, facial expressions convey information about what emotion is currently experienced by a target, which in turn affects how the target is perceived and what behavioral tendencies are elicited in the observer [1], [2], [3]. Correspondingly, substantial research has revealed that correctly recognizing another's emotion is positively associated with successful social functioning [4], [5], [6] and negatively related to loneliness [7]. The relevance of correctly recognizing emotions in others' faces renders the identification of factors determining facial emotion recognition highly important.

In this vein, the present research links facial emotion recognition to broad and basic motivational orientations, namely regulatory focus [8]. Studying the impact of regulatory focus on facial emotion recognition seemed to us particularly worthwhile, because regulatory focus introduces a fundamental distinction between two motivational strategies that have been shown to affect an enormous range of phenomena ranging from basic motivational mechanisms [9] to intergroup relations [10]. Moreover, regulatory focus is likely to affect emotion recognition, because regulatory focus has been shown to affect performance in well-learned tasks [11], [12] that are performed (almost) without monitoring (i.e., attention to task execution). This clearly applies to facial emotion recognition as it is very routinely performed [13] and one might even ague that it is innate [14].

Beyond the impact of regulatory focus on emotion recognition, our research also investigated *how* the two different motivational orientations exert their influence by examining how regulatory focus affects eye movements indicative of visual attention allocation. Specifically, the eagerness strategy inherent in a promotion focus should encourage rapid shifts of attention at encoding whereas the vigilance strategy inherent in a prevention focus should encourage more sustained attention at encoding. In turn, these attentional strategies would be expected to respectively support or undermine successful facial emotion recognition.

In sum, the current research sought to provide evidence that regulatory focus is a motivational determinant of facial emotion recognition because of regulatory focus's impact on visual attention allocation during face encoding. The present research is thus not only the first to link regulatory focus and emotion recognition, but it is also the first to study the impact of regulatory focus on visual processing by means of eye tracking in the context of emotion recognition.

### Regulatory Focus and Task Performance

Regulatory Focus Theory (RFT, [8]) suggests that there are two basic modes of self-regulation (regulatory foci) by which people pursue their goals. When employing a *promotion focus*, people are guided by the need for growth and accomplishment and pursue ideals and aspirations by eagerly striving towards them. The eagerness strategy triggered by a promotion focus is best described as continuous striving to achieve successful outcomes (i.e., to make hits as regulatory focus researchers have put it) by using as many opportunities to take action as possible thus avoiding errors of omission. When employing a *prevention focus*, people are guided by the need for security and safety and pursue the fulfillment of responsibilities by strategically avoiding possible failure or risks in goal attainment. The vigilant strategy elicited by a prevention focus seeks success by acting cautiously to avoid errors of commission (for evidence of the strategic implications of regulatory focus, see [15]). Both foci vary situationally as well as chronically between individuals.

Regulatory Focus Theory possesses substantial explanatory power and has successfully elucidated diverse areas of human behavior. Although the theory was articulated to explain intra-individual processes related to goal pursuit [8], [16], [17], its application has since then yielded new and exciting insights into how intra-individual self-regulatory strategies affect inter-individual (e.g., romantic relationships [18], [19], interaction with partners [20], leadership [21]) as well as inter-group phenomena (e.g., group identity [22], stereotype threat [12], social discrimination [23]).

Particularly relevant findings for the current research question address the impact of regulatory focus on well-learned tasks that are to be performed as quickly and correctly as possible [11], [12]. Well-learned tasks indicate tasks that are routinely executed and do not require monitoring (i.e., attention to task execution), because they have highly frequently been performed and are overlearned. Because of its emphasis on avoiding errors of omission, a promotion focus prompts people to move quickly from one task to the next or from one stimulus to another in their immediate context. To be more precise, the tendency to avoid errors of omission requires scanning the environment for other opportunities. Hence, visual attention is captured by task cues for relatively brief periods and frequently directed towards other cues that might indicate other opportunities. This provides optimal conditions to perform routine tasks that rely on automatic or associative processing and that might even suffer from substantial monitoring or elaboration. For example, a promotion focus facilitates the detection of context free errors during a proof reading task (e.g., typos) relative to a prevention focus because context free errors can be detected with less close reading and quick progression to the next cue [11]. Because of its emphasis on avoiding errors of commission, a prevention focus prompts people to examine task elements closely and to scrutinize behavior for mistakes as it unfolds. For example, relative to a promotion focus, a prevention focus facilitates the detection of context dependent errors during a proof reading task (e.g., the wrong form of ‘their’) that would be missed without careful scrutiny of easily read texts.

In addition, the attentional strategies associated with a promotion focus and a prevention focus also play out in visual attention. The eager striving not to miss an opportunity in a promotion focus leads to a broader attentional scope than the thorough processing of task details in a prevention focus. Recent research indeed provided evidence for this prediction showing that a promotion focus leads to a broader scope of visual attention than a vigilant prevention focus using eye tracking [24].

In the context of complex but well-learned tasks, these strategies (moving quickly between cues vs. scrutinizing task elements for mistakes) have clear implications for task performance. People become more capable as tasks become better learned precisely because they no longer have to carefully consider individual task elements separately. Rapid integration of task elements is a hallmark of expertise whereas careful serial processing of task elements is more typical of a task novice [25], [26]. Indeed, a large part of the phenomenon of “choking under pressure” results from scrutiny of well-learned behavior that is best left to unfold automatically [25].

Taken together, these findings suggests that the attentional strategy prompted by a promotion focus (i.e., moving quickly between task elements) is likely to facilitate performance on a well-learned task compared to a prevention focus [11].

### Regulatory Focus and Visual Attention in Facial Emotion Recognition

Facial recognition is usefully treated as an exceptionally well-learned skill [13]. Faces are much better detected than comparable non-face stimuli [27]. Moreover, facial emotion recognition can be non-consciously performed given that facial information, including emotions, can be extracted from faces in a few hundred milliseconds or less (e.g., *56* ms, [28], *67* ms, [29], *200* ms, [30]). Facial recognition is thus routine and automatic.

This does not imply that human facial processing is not learned rather than innate. Many facial emotional expressions are recognized cross-culturally [31] and recent findings indicate that infants are able to produce facial expressions in the womb [14] suggesting that it might even be innate. Indeed, face recognition has a long developmental trajectory in which it is refined with exposure to ever increasing numbers of faces [32].

Regardless of its developmental origins, facial emotion recognition appears to be automatized in the sense that monitoring of facial emotion recognition efforts is not required for successful recognition outcomes. In fact, there is evidence that such monitoring impairs recognition performance [33].

Based on this evidence, we predict that regulatory focus will affect facial emotion recognition such that a promotion focus will facilitate facial emotion recognition compared to a prevention focus.

Additionally, our theoretical framework specifies the mechanism by which regulatory focus should affect facial emotion recognition. An eager motivation to avoid errors of omission (i.e. promotion focus) leads to rapids shifts of visual attention between elements of the task at hand and other cues in the context. These rapid shifts of attention should facilitate performance on the routine task of facial emotion recognition.

This mechanism highlights ambiguities around the impact of a prevention focused attentional strategy on facial emotion recognition. Regulatory focus theory argues that chronic promotion and prevention focus are independent concepts that each affect a certain set of outcomes (e.g., global vs. local processing, [34]). In this vein, Förster and colleagues [11] argued that a promotion focus mainly affects performance on well-learned tasks whereas a prevention focus has a stronger impact when more elaborate processing is required. For routine tasks like emotion recognition in faces, this theoretical claim implies that a promotion focus and the resulting visual attention strategy should have a stronger impact than a prevention focus and the resulting visual attention strategy. Unfortunately Förster and colleagues do not provide clear evidence for this claim as the crucial study lacks a control condition (Exp. 4). Due to the lack of empirical evidence, it is hard to predict *a priori* whether promotion focus, prevention focus, or both would drive the predicted effects.

Our prediction is mainly derived from the regulatory focus literature, but is also consistent with the existing facial emotion recognition literature. Expertise in face recognition and facial emotion recognition is underpinned by holistic encoding, processing the components of a face in parallel and as a gestalt whole rather than as serial elements [35], [36], [37], [38], [39], [40]. The visual attention strategy involving rapid shifts of attention associated with a promotion focus is consistent with such holistic encoding, whereas the sustained scrutiny of task elements associated with a prevention focus is not.

We investigated these ideas in two studies. In Study 1, we manipulated regulatory focus and observed the effects on facial emotion recognition. In Study 2, we measured chronic regulatory focus, assessed gaze fixation duration as an index of visual attention, and observed the relationships with facial emotion recognition.

### Ethics statement

Both Studies 1 and 2 were approved by the Ethics Commission of the Knowledge Media Research Center (Tübingen, Germany) and all participants in both studies have given written informed consent prior to participating in the studies.

## Study 1

### Method

#### Participants and Design

Ninety-five undergraduate students at a German university (57 women, *M_age_* = 25.27 years, *SD* = 3.26, range: 20–37) participated in an experiment with two conditions (Regulatory Focus: prevention focus vs. promotion focus). All participants received 8 Euro (approximately 10 $) for compensation.

#### Procedure

Participants were recruited for a study package on ‘person perception’. Groups of up to six individuals participated during one experimental session. Upon arrival in the laboratory, participants were seated in semi-private cubicles. All further information was provided by computer.

The experimental session started with the regulatory focus manipulation. Participants recalled two promotion-type successes and one promotion-type failure or two prevention-type successes and one prevention-type failure [41], see also [21], [42], [43]. Specifically, in the promotion focus condition participants had to recall and write down a few lines each regarding (a) a situation in which they “felt like they made progress towards being successful in their life”, (b) a situation in which they “felt like they failed to make progress towards being successful in their life” and finally (c) a situation in which “compared to most people, they were able to get what they wanted out of life”. Likewise, in the prevention focus condition, participants had to recall and write down a few lines each regarding (a) a situation in which “being careful enough had prevented them from getting into trouble”, (b) one situation in which “not being careful enough had got them into trouble” and (c) one situation in which “they acted in a way that nobody would consider objectionable”. By remembering both success and failure situations in both conditions, we aimed at keeping affect constant across the two regulatory focus condition, thereby assuring that differences between conditions are likely due to the different content participants recalled, promotion and prevention strategies.

Afterwards, participants worked on an emotion recognition task using pictures taken from the Diagnostic Analysis of Nonverbal Accuracy (DANVA2, [44]), a well-established measure of facial emotion recognition [45], [46], [47], [48]. This task involved identifying the emotions expressed in 24 pictures of adult faces displaying happiness, sadness, fear, or anger in varying intensities. Stimulus faces were presented for three seconds in the center of the screen (horizontal visual angle: 26.43°, vertical visual angle: 16.63°) and response options remained onscreen until participants had answered.

### Results and Discussion

As predicted, promotion focused participants (*M* = .77, *SD* = .09) correctly identified a larger proportion of facial emotions than did prevention focused participants (*M* = .72, *SD* = .13), *t*(88)  = 2.05, *p* = .043, *d* = .44, CI_d,95%_ =  [.03;.85]. This result provides initial empirical support for the hypothesis that promotion focused motivational orientation facilitates facial emotion recognition relative to prevention focused motivational orientation, thereby indicating a relation between observer's regulatory focus and facial emotion recognition performance. We believe this effect occurred because of the relation between regulatory focus and task-related visual attention. However, as Study 1 provides no direct evidence supporting this hypothesis we conducted a second study aimed to fill in this gap.^1^

## Study 2

Study 2 replicated and extended Study 1 in three key ways. First, Study 2 operationalized regulatory focus as a chronic individual difference. Convergent results across different operationalizations would support the robustness of the findings reported in Study 1.

Second, Study 2 directly measured visual attention using eye tracking. Specifically, we indexed visual attention with mean fixation duration. Mean fixation duration is a well-established indicator of online perceptual and cognitive processing that has been applied in a wide range of psychological research areas (e.g., cognitive psychology, developmental psychology, or media psychology, [55], [56], [57], [58]). Mean fixation duration indicates how much time is devoted to processing particular pieces of information before attention shifts [59], [60]. It is computed by dividing dwell times by number of fixations which corresponds to how long individuals fixate one location before ‘jumping’ their gaze to another (see below for further details).

Low values on this indicator capture the wide attentional net associated with a strong promotion focus. The conflict between working on a task element and searching for other opportunities (i.e., avoiding errors of omission) results in short fixations on task elements alternating with inspection of the task environment. Critically, this conflict could not be extracted by looking at either of the components of mean fixation duration (number of fixations or dwell time) in isolation from one another. This direct online measurement of visual attention thus allowed us to evaluate the hypothesis that individuals with a stronger promotion focus would show rapid shifts of attention between target faces and distracting cues in the environment. In turn, these rapid shifts of attention were expected to be related to better performance because a wide attentional net reflects the most appropriate strategy for routine tasks like emotion recognition [25], [26].

Third, target facial stimuli were presented in the context of other materials because the distinction between an eager strategy aimed at avoiding errors of omission and a vigilant strategy aimed at avoiding errors of commission should be clearer if there are stimuli present that might plausibly be omitted from visual processing.

### Method

#### Participants and design

Forty-six undergraduate students at a German university (30 women, *M*_age_ = 24.50 years, *SD* = 4.55, range: 19–39) with normal or corrected-to-normal vision participated in a study on ‘eye movements in person perception’. Chronic promotion and prevention focus were assessed as continuous predictors. Participants' eye fixations during an emotion recognition task and performance on the emotion recognition task served as process and outcome variables, respectively. All participants received 8 Euro (approximately 10 $) for compensation.

#### Procedure

The procedures of Study 2 paralleled Study 1 with the following exceptions. The study was run in individual sessions. Upon participants' arrival in the laboratory, they were seated in front of a 22 inch monitor in a distance of approximately 25 inches. The eye tracking system was then calibrated using a nine-point system. The emotion recognition task [45] was adapted for use with the eye tracking paradigm by starting every trial with a fixation cross in the center of the screen followed by an emotion stimulus in one of the four corners of the screen or in the center of the screen. The pictures were reduced in size by one third and overlaid on cut-outs of newspapers pages that covered the whole screen (visual angles for the DANVA2 target pictures: horizontal angle: 10.39°, vertical angle: 7.46°; visual angles for the complete picture (distractor newspaper picture covering most of the screen including the target picture): horizontal angle: 36.18°, vertical angle: 19.16°).

**Figure 1:**
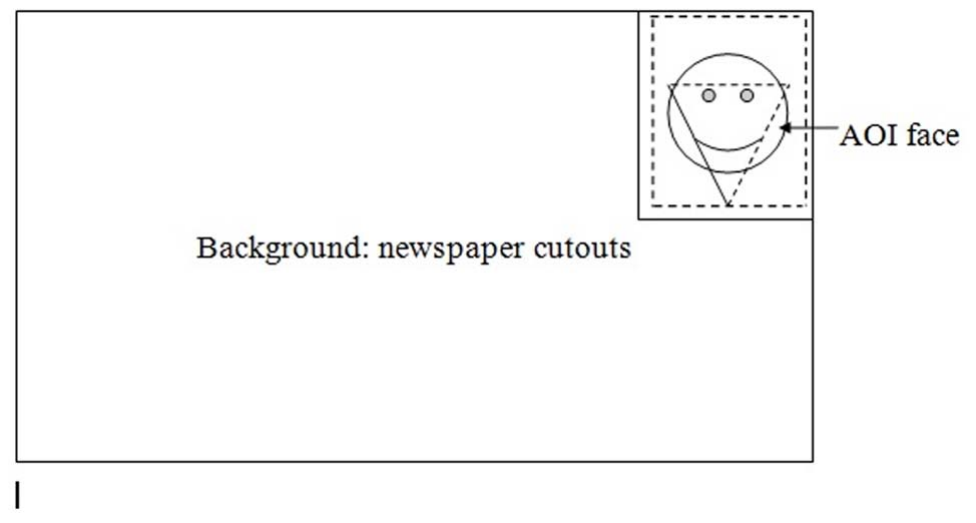
Schematic example of stimulus picture (Study 2).

These backgrounds were intended to mimic face perception in cluttered unpredictable visual environments. We used 24 different newspaper extracts for the 24 pictures of the DANVA2. Hence, each DANVA2 picture was paired with a different newspaper background. All newspaper extracts were carefully chosen to contain a picture, text, and a heading of neutral to mildly positive content. Moreover, we tested these newspaper articles used as distractor background pictures in a different sample. Results of this test indicated that the pictures did not to contain strong emotional or regulatory focus related content (see Figure 1 for details).

After working on the emotion recognition task, participants filled out a questionnaire assessing regulatory focus as an individual difference (adapted from [21], [61], [62]). Validation studies have shown that the two subscales of this regulatory focus measure correlate closely (.50<rs<.60) with the respective subscales of the other most widely used measure of regulatory focus, the Regulatory Focus Questionnaire [41]. The prevention focus subscale (e.g., “When making important decisions, security is a fundamental criterion for me.”, “At work and in my studies being accurate is very important.”, “I am not a cautious person.” (reversed), α = .61) consisted of eight items, the promotion focus subscale (“I strive for success in my life”, “I strive for progress.”, “I want to achieve a great deal.”, α = .67) consisted of 12 items. All items used a seven-point Likert scale, ranging from “1 =  *does not apply to me at all*” to “7 =  *completely applies to me*”.

#### Eye Tracking

Eye movements were recorded using a SMI RED eye-tracker (SensoMotoric Instruments), a standalone remote eye tracking device with an accuracy of 0.5 degrees and a sampling rate of 50 Hz. The minimum fixation duration was set to 100 ms with a fixation radius of 100 pixels [57], [63]. These event detection settings were determined based on first plotting the raw data and then analyzing the raw data using different event detection settings [64]. The settings with the closest match to the raw data were chosen (i.e., where sequences of gazes in close proximity were correctly identified as being one fixation and more distant gazes were identified as belonging to a new fixation). Stimulus material was presented using Experiment Center 2.4 and eye movements during the emotion recognition task were analyzed with BeGaze 2.4 (http://www.smivision.com). For the analysis of the eye tracking data, the raw data were first aggregated into fixations, that is, events during which eye gaze was maintained on a single location and information uptake could take place.

In a second step, we defined Areas Of Interest (AOIs) to differentiate eye movements that were plausibly related to emotion encoding from irrelevant eye movements. The critical AOI covered the most relevant facial features for detecting the emotional expressions under consideration, namely the eyes (including eyebrows) and the mouth of the displayed faces ([49], [65], [66], see Figure 1 for detail). The remainder of the display was classified as a second irrelevant AOI. More precisely, this irrelevant AOI covered the distractor newspaper background in which the target facial emotion expressions were embedded.

To determine our process variable, we calculated mean fixation duration separately for the two AOIs for each picture by dividing the time spent looking at a given AOI (i.e., dwell time) by the number of fixations on that AOI (see Table 1 for absolute values of mean fixation duration, attentional dwell time, and number of fixations on both AOIs). Due to central fixation bias [67] the four pictures presented at the center of the screen (the location of the fixation cross) produced very little variance in eye gaze behavior so we did not include those stimuli.^2^

**Table 1:** Means and standard deviation of mean fixation durations, attentional dwell times, and number of fixations for the two AOIs in Study 2.

	Critical AOI		Irrelevant AOI	
	*M*	*SD*	*M*	*SD*
Mean fixation duration	371.65	134.96	472.33	149.38
Attentional dwell time	503.22	300.70	1564.96	387.72
Number of fixations	6.05	5.52	75.09	16.60

### Results and Discussion

Regression diagnostics indicated two cases that were disproportionately influential (based on large studentized deleted residuals, 2.77 and −2.15, and on outlying values for Cook's D,.31 and.19). We opted to exclude these cases from the analyses although their inclusion does not substantially change the outcomes.

The current study tested whether a chronic promotion focus fosters facial emotion recognition and whether a chronic prevention focus hinders facial emotion recognition. In addition, this study served to test whether the impact of regulatory focus on facial emotion recognition is mediated by task-related patterns of attention allocation, as indexed by mean fixation duration.

A multiple regression analysis with emotion recognition as the criterion and the two regulatory foci as predictors revealed that a stronger promotion focus predicted enhanced emotion recognition, (*b* = .06, *SE* = .02, *p* = .001, CI_b,95%_ =  [.024;.090]), whereas a chronic prevention focus was unrelated to emotion recognition (*b* = .01, *SE* = .01, *p* = .662, CI_b,95%_ =  [−.022;.035]).

Analyzing the effect of Regulatory Focus on visual attention, a multiple regression analysis with mean fixation duration as the criterion revealed that a stronger promotion focus predicted shorter mean fixation durations (*b* = −53.79, *SE* = 23.34, *p* = .026, CI_b,95%_ =  [−100.96; −6.62]) on the critical AOI, whereas prevention focus did not relate to mean fixation duration (*b* = 23.59, *SE* = 20.07, *p* = .240, CI_b,95%_ =  [−16.62; 64.52]). Neither promotion nor prevention focus were related to mean fixation durations or proportion of time spent on the irrelevant AOI (i.e. the AOI involving the distractor background; *mean fixation duration:* promotion focus: *b* = −9.27, *SE* = 27.59, *p* = .738, CI_b,95%_ =  [−64.99; 46.44]; prevention focus: *b* = .76, *SE* = 23.59, *p* = .974, CI_b,95%_ =  [−46.88; 48.40]; *proportion of time:* promotion focus: *b* = −.002, *SE* = .099, *p* = .986, CI_b,95%_ =  [−.198;.202]; prevention focus: *b* = −.102, *SE* = .089, *p* = .183, CI_b,95%_ =  [−.299;.059]).^3^

To test whether promotion focus improved facial emotion recognition through its effects on attention allocation, we estimated the indirect effect of a promotion focus on facial emotion recognition through fixation duration. To this end we applied bootstrapping using the SPSS macro provided by [68]. The resulting confidence interval did not contain zero (1000 re-samples, *b* = .0129, CI_b,95%_ =  [.0024;.0383]) indicating a significant effect of promotion focus on facial emotion recognition via task-related attention allocation (as indicated by shorter fixation duration). We controlled for prevention focus in this bootstrapping analysis.

These results support our hypothesis that regulatory focus affects facial emotion recognition through its effects on visual attention. Specifically, promotion focus fostered accuracy in facial emotion recognition through rapid shifts of attention away from the task AOI.

One might expect that, in addition to affecting performance, chronic regulatory focus would affect reaction times in the DANVA2. As a promotion focus is associated with eagerly moving on with the task at hand and a prevention focus is associated with caution a promotion focus might lead to faster decisions and a prevention focus might lead to slower decisions. However, the time available to participants to process each facial expression was limited and constant. Such constraints enhance performance effects at the expense of reaction time effects [69], [70]. In addition, we have doubts about whether response time effects should be expected in the first place. Free of constraint, prevention focused individuals should indeed spend more time on the task than promotion focused individuals. However, the tendency of those in a promotion focus to scan the environment to avoid errors of omission might work against their speed advantage. This might explain why regulatory focus did not affect the speed with which participants moved from trial to trial (promotion focus: *b* = −130.45, *SE* = 219.07, *p* = .555, CI_b,95%_ =  [−572.86; 311.97]; prevention focus: *b* = −190.72, *SE* = 195.37, *p* = .335, CI_b,95%_ =  [−585.29; 203.84]).

It might also seem surprising that the eager strategy to avoid errors of omission did not also lead to longer mean fixation durations on the distracting materials. However, predictions about gaze behavior for the irrelevant AOI are not straightforward. Logically, if participants look away from the task relevant facial expression, as predicted by a promotion focus, they should be looking at the irrelevant AOI more often. This gaze behavior would be captured by number of fixations, one component of mean fixation duration. The relationship between regulatory focus and dwell time for the irrelevant material, the second component of mean fixation duration, is not theoretically specified, however. Neither promotion nor prevention focused participants would have particular reason to dwell on irrelevant material. As both number of fixations and dwell time are necessary to capture the attentional strategies predicted by regulatory focus theory, meaningful information about the attentional strategies associated with regulatory focus cannot be extracted from the irrelevant AOI.

Overall, the present results replicate and extend the findings of Study 1 by using an alternative operationalization of regulatory focus and by providing empirical support for the underlying mechanism.

## Discussion

In two studies we linked observer motivation in the form of regulatory focus to facial emotion recognition. In Study 1, a situationally activated promotion focus enhanced facial emotion recognition relative to a situationally activated prevention focus. In Study 2, a stronger dispositional promotion focus fostered facial emotion recognition through task-related patterns of visual attention allocation, as indicated by shorter duration of fixations on a target person's face. These results empirically link a motivational concept that is broadly influential on information processing and social behavior - regulatory focus - with facial emotion recognition performance and also identify visual attentional strategies underlying this effect.

More precisely, the present findings indicate that the motivational nature of a promotion focus orientation elicits an information processing strategy that is beneficial for facial emotion recognition. The eagerness and aversion to errors of omission inherent in a promotion focus lead to rapid shifts of visual attention during encoding and thereby to relatively short encoding intervals (as opposed to sustained scrutiny during encoding in a prevention focus; [11]). This visual attentional strategy facilitates facial emotion recognition because it fits the task monitoring strategies of highly automatized tasks. In this work, we measured visual attentional strategies online by assessing mean fixation duration via eye tracking. The stronger an individuals' promotion focus the more rapidly they shifted their attention during encoding, which was in turn associated with enhanced emotion recognition.

We observed a link between a promotion focus, visual attention allocation, and facial emotion recognition, but not between a prevention focus and visual attention allocation or facial emotion recognition. These results are in line with the theorizing that a promotion focus but not a prevention focus exerts impact on performance in routine tasks [11]. Still, a prevention focus would be expected to exert patterns of visual attention that undermine facial emotion recognition. This missing relationship might be explained by multiple sources of influence on facial emotion recognition exerted by prevention focus. One possibility is that prevention focus is related to increased interdependence [71] and interdependence, in turn, is related to enhanced perspective taking [72] – an other-oriented concept that shares substantial communalities with facial emotion recognition [73]. However, a link between visual attention allocation and interdependence has, to our knowledge, not been specified and other findings indicate interdependence to be negatively related to the decoding of emotional expressions [74], [75].

Another possibility is that the links between promotion focus and visual attention and between prevention focus and visual attention are actually asymmetrical. In the athletic performance literature, attentional scrutiny that undermines performance is explicitly conscious [25], [26]. Similarly, the tasks used to establish attentional differences between regulatory foci in Förster and colleagues' work ([11], e.g., proof reading) were amenable to conscious inspection. In contrast, creating conscious interference with face processing requires careful and indirect manipulation (e.g., verbal overshadowing [33]). The tendency for promotion focus to encourage “going with the flow” is consistent with unconscious automatized action whereas the tendency for prevention focus to encourage scrutiny might be dependent on subjecting action to conscious examination. Actions that are so automatized as to be difficult to make conscious, like facial emotion recognition, might thus be insulated against the influence of prevention focus.

The present work's use of eye-tracking to assess visual attention allocation reflects an advanced method in the emotion recognition literature that draws on innovations developed to understand non-emotional face perception [57]. Earlier work on facial emotion recognition has mainly relied on indirect manipulation of processing through priming [38] or altering facial stimuli [36], [39], [40], [76]. Assessment with eye-tracking allows processing to be indexed *online*, as it unfolds during facial emotion recognition. The present research thus helps to validate an important new method in the study of visual attention allocation and facial emotion recognition [77].

Our predictions and measurement derive from the regulatory focus literature and from the literature on routine tasks. We have thus referred to cognitive processing and attention allocation in terms that generalize across these tasks. Within the face processing literature, expertise is usually coupled with terms like holistic, global, or configural processing. A visual attentional strategy that quickly moves between task elements is completely consistent with the ideas of holistic, global, or configural processing. In fact, mean fixation duration is closely related to one of the key elements in an index of global processing [57]. Although the details of our methodology do not allow computation of a full index of global processing (emotional faces we were relatively small in our paradigm, 100 pixels at their broadest point; see Figure 1 for an illustration), we view it as likely that the visual attentional strategy we describe corresponds to a holistic, global, or configural encoding strategy.

The present findings also relate to work on affect and attentional scope [78]. Indeed, empirical findings indicate similar effects of regulatory focus and positive/negative affect on cognitive processes such as attentional scope [24], [78] or creative cognition [79], [80]. However, in our view there are several reasons to believe that affect does not play an important role in the present findings. On a theoretical level, Regulatory Focus Theory [8] asserts that promotion and prevention focus increase the sensitivity for gains and losses, respectively. But prevention and promotion focus are not positive or negative affect states. Instead, they correspond to different strategies applied during goal attainment – eagerly aiming to make hits in a promotion focus and avoiding errors in a prevention focus. Hence, promotion and prevention focus can be activated independent of any information about success or failure. Positive or negative self-relevance is, however, a necessary precondition for affect. Thus promotion and prevention focus are not closely linked to certain affective valences, but can be understood as states of cognitive preparedness to process gain and loss signals leading to certain strategic inclinations (see also [43], for further details). In fact, research indicates that both promotion and prevention focus are related to both positive and negative affective states. Promotion focus is related to emotions such as happiness or dejection and prevention is related to relief or anxiety [53].

With regards to the specific methodologies of the two studies reported here, we took care to keep affect constant across manipulation and measurement regulatory focus. Our manipulation in Study 1 involved recall of both successes and failures for both promotion and prevention focus. Similarly, the scale we used in Study 2 counterbalances items referring to promotion and prevention successes and failures (see [62] for more details on the items), thereby avoiding an assessment of regulatory focus that is confounded with affect.^4^

Our findings open new avenues for research on the antecedents and consequences of emotion recognition. For example, differences in emotion recognition between a promotion and a prevention focus might help to explain findings about regulatory focus at the interpersonal level. Righetti, et al. [19], [20] repeatedly found stronger interpersonal effects of a promotion focus than of a prevention focus. This might actually be due to the better emotion recognition performance in a promotion focus. Likewise, recent research has shown that a promotion focus is associated with transformational leadership behavior [21], a concept that is also associated with enhanced emotion recognition [83]. Finally, individuals high in power are better at facial emotion recognition, than are low power-individuals [48], but see also [45]. Our findings link these observations as high power is associated with a promotion focus whereas low power is associated with a prevention focus [84], [85].

Considering the association between regulatory focus and other psychological constructs, for example approach and avoidance tendencies [86], [87], also raises other exciting research questions. It would be interesting to examine whether approach tendencies foster facial emotion recognition compared to avoidance tendencies. On the strategic level, promotion focus implies eagerly *approaching* a positive end state, thereby making task-related attention allocation (indicated by shorter fixation duration) as a consequence of approach motivational orientation also more likely. At the same time, perspective taking (a concept related to facial emotion recognition) is facilitated by avoidance rather than approach motivational orientation [88].

The present research also has potential applied value. Our findings suggest that individuals for whom the correct identification of expressed emotions is highly relevant (e.g., psychotherapists, team leaders, or teachers) will be best served by striving to maximize successes (i.e., be promotion-focused) rather than to minimize mistakes (i.e. be prevention-focused). In a similar vein, the present results suggest that when instructing individuals to correctly identify facial emotion expressions, one should consider the framing of this instruction because specific task instructions can induce either a promotion or a prevention focus [15].

To conclude, motivational orientation is an important but thus far neglected influence on facial emotion recognition. Regulatory focus provides a well-grounded starting point for the study of observer motivation on emotion recognition, but is only the beginning of a potentially rich addition to the understanding of emotion recognition. It is our sincere hope that the foundations laid in this work will prove generative for future research.

### Footnotes

1. Based on literature that suggests women better detect facial expressions of emotion than do men [49], [50], we also included gender as factor in our analyses. However, we found neither a main effect of gender on facial emotion recognition nor moderation of the relation between regulatory focus and facial emotion recognition by gender (both *p*s>.30). In our understanding, the well-established gender-effect in emotion recognition might have been overruled by the regulatory focus manipulation as gender and regulatory focus are (complexly) related [51]. In addition, the vanishing of ‘classical’ gender roles might also contribute to reduced gender differences in emotion recognition [52]. Similarly, RFT literature suggests that the relationship between regulatory focus and (facial) emotion recognition might be moderated by the type of emotion. RFT implies specific emotional consequences for success and failure under different regulatory foci. Happiness and sadness are respectively associated with a promotion focus and quiescence and agitation are respectively associated with a prevention focus [22], [53]. Furthermore, research indicates that positive emotional words are better detected and remembered under a promotion focused motivational orientation, whereas negative emotional words are better detected and remembered under a prevention focused motivational orientation [54]. However, including type of expression as experimental factor in our analyses did not reveal any moderating effect on the relationship between regulatory focus on facial emotion recognition (*p*<.30). The same was true for Study 2. In our view, the main difference between our research and the work mentioned above [53], [54] is that we used pictorial stimuli and not semantic stimuli (i.e., emotional words) which are most likely differently processed compared to pictorial material. Pictorial expressions, for instance, but not semantical stimuli allow for mimicry. Furthermore, we also assessed reaction times to the 24 pictures in this study. However, our regulatory focus manipulation did not affect reaction times (*p*>.60).

2. When these four trials are included in analysis, both predictors, promotion focus and prevention focus, were reduced in strength so that they no longer significantly predict mean fixation duration. These findings are not surprising given that these trials presented the faces and initial fixation cross at the same location on screen. The necessity for visual search was thus reduced in turn leaving less room for participants' regulatory orientation to exert influence on their attentional strategies during the task.

3. For the sake of completeness, we also analyzed the impact of promotion and prevention focus on dwell time as well as on fixation numbers separately. Analyses reveal that the effect of promotion and prevention focus on the number of fixations within the critical AOI is equally low (promotion focus: β = −.139, *p* = .374; prevention focus: β = −.142, *p* = .362). Both promotion and prevention focus tend to be negatively associated with numbers of fixation. In contrast, promotion focus tends to be associated with shorter dwell times whereas prevention focus is shows no descriptive relationship with dwell time (promotion focus: β = −.212, *p* = .183 prevention focus: β = −.041, *p* = .794). Importantly the visual attentional strategy under discussion involves the relationship between fixation and dwell time. In isolation, neither component captures the visual attentional strategy expected to be associated with a promotion or a prevention focus. Analysis of our main index, mean fixation duration, is thus substantially more informative than is analysis of the components in isolation from one another.

4. This notion is further supported by unpublished data from our lab [81]. We assessed individuals' regulatory focus using the same scale as in Study 2 and assessing affective state using a short version of the Positive And Negative Affect Schedule (PANAS, [82]). Positive affect correlated with both, promotion (*r* = .27, *N* = 63, *p* = .034) and prevention focus (*r* = .34, *N* = 63, *p* = .006). Thus, it is not likely that affect is responsible for the findings of Study 2.
